# Local acceleration of coastal flood risk in response to relative sea level change

**DOI:** 10.1038/s41598-025-13021-3

**Published:** 2025-07-26

**Authors:** Ryan Paulik, Rebecca Welsh, John Powell

**Affiliations:** 1 Earth Sciences New Zealand, 301 Evans Bay, Greta Point, Wellington, 6021 New Zealand; 2 AtkinsRéalis, London, England

**Keywords:** Natural hazards, Climate sciences

## Abstract

Coastal communities are expected to experience an increased episodic flooding threat as sea levels rise during the 21st century. While global sea level rise (SLR) is a significant contributor, local processes such as vertical land motion (VLM) influence the flooding threat timing and magnitude. This study estimated building financial losses for extreme sea level-driven flooding and relative sea level (RSL) change in New Zealand. Financial losses were calculated for individual building objects over a future 100-year period using SLR projections for medium confidence Shared Socio-economic Pathway (SSP) scenarios 2–4.5 and 5.8–5, with local VLM. Local VLM increased national 100-year coastal flooding losses by up to 15% at 2100 compared to SLR alone, and bringing forward the expected end-of-century losses by 10–12 years. At subnational levels, annual losses between 2050 and 2100 could occur up to 25 years earlier from downward land motion and 19 years later from upward motion. These findings highlight the importance of including VLM in coastal flood risk assessments to inform risk mitigation location and timing under changing RSLs.

## Introduction

Rising sea levels this century are expected to increase the magnitude and frequency of socio-economic impacts from episodic extreme sea-levels (ESL). Global mean sea level rise (GMSLR) driven by climate change under low-emission scenarios, puts at least 190 million people at risk to permanent or episodic flooding by the year 2100, increasing to 630 million under high-carbon emission scenarios^1^. Populations residing in major coastal cities could experience annual financial losses of approximately US$52 billion by 2050 and surpass US$1 trillion if no mitigation measures are taken^2^. In Europe, without investment in mitigation interventions^3^direct economic loss from coastal flooding could respectively grow to over €209 billion and €1268 billion under ‘sustainability’ and ‘fossil fuelled’ Shared Socio-Economic Pathways (SSP)^3,4^. These futures that coastal populations face require local adaptation strategies to mitigate socio-economic consequences under changing climate conditions.

Rising mean sea levels will exacerbate localised episodic flooding from ESLs caused when tides, low atmospheric pressure and wind-driven waves combine to produce storm-tides^5^. Future GMSLR projections under different SSPs show an expected acceleration after 2050, relative sea level (RSL) change can be accelerated by vertical land motion (VLM)^6–9^. Downward land motion can bring forward permanent land inundation timing by years or decades^9,10^ and exacerbate exposure to high-impact coastal flood events^11^. Coastal flood impacts from combined GMSLR and VLM effects are often evaluated at subnational levels^11–14^. These studies demonstrate that VLM is not continuous along coastlines and could shift local flood risk regimes toward more frequent and higher magnitude impacts in the future. Such local effects can be overlooked in large-scale (i.e., national or supra-national) coastal flood risk assessments, particularly when GMSL alone or a uniform VLM rate is considered.

Variable future RSL change rates over short coastline distances demand an accurate representation of tangible flood-exposed assets (e.g. buildings, infrastructure network components) in coastal flood risk assessments. A well-practised risk evaluation approach is calculating the average annualised loss (AAL) (also known as expected annual damage (EAD)) for exposed assets^15^. The metrics represent expected economic losses for assets in a single year and are used to evaluate and optimise mitigation intervention cost-benefits. In large-scale coastal flood risk assessments AAL is calculated using continuous land use maps representing location-specific assets^4^. This approach aggregates assets into common typologies and economic unit values over areas ranging between 100 s and 1000 s of square meters. Space-varying flooding hazards over a few meters necessitate economic loss evaluation for individual assets-at-risk. While several nationwide studies^16,17^ enumerate exposure to future coastal flood regimes, AAL evaluation for high-value assets such as buildings is limited^18^. Risk information at the asset-level is critical for location-based prioritisation of hazard mitigation inventions (e.g., levees, nature-based solutions) and risk transfer management (e.g., insurance, mortgages).

This national study estimates financial losses from building exposure to episodic coastal flooding and relative sea level (RSL) change in New Zealand. We use a nationwide RSL projection database^19,20^ to evaluate loss changes over the next 100-years in response to global mean sea level rise (GMSL) and local vertical land motion (VLM). Financial loss timing and magnitude were analysed at building level for projected RSL change under shared socio-economic pathways (SSP) 2-4.5 and 5-8.5 medium confidence scenarios. We report on national, regional and territory-level financial losses for 100-year annual recurrence interval (ARI) flooding events and average annualised loss (AAL), important metrics for coastal hazard management as stipulated in the New Zealand Coastal Policy Statement^21^. The analysis identifies spatiotemporal shifts in expected losses in response to local VLM direction.

## Results and discussion

### Vertical land motion effects on financial losses for 100-year annual recurrence interval coastal flooding

**Fig. 1:**
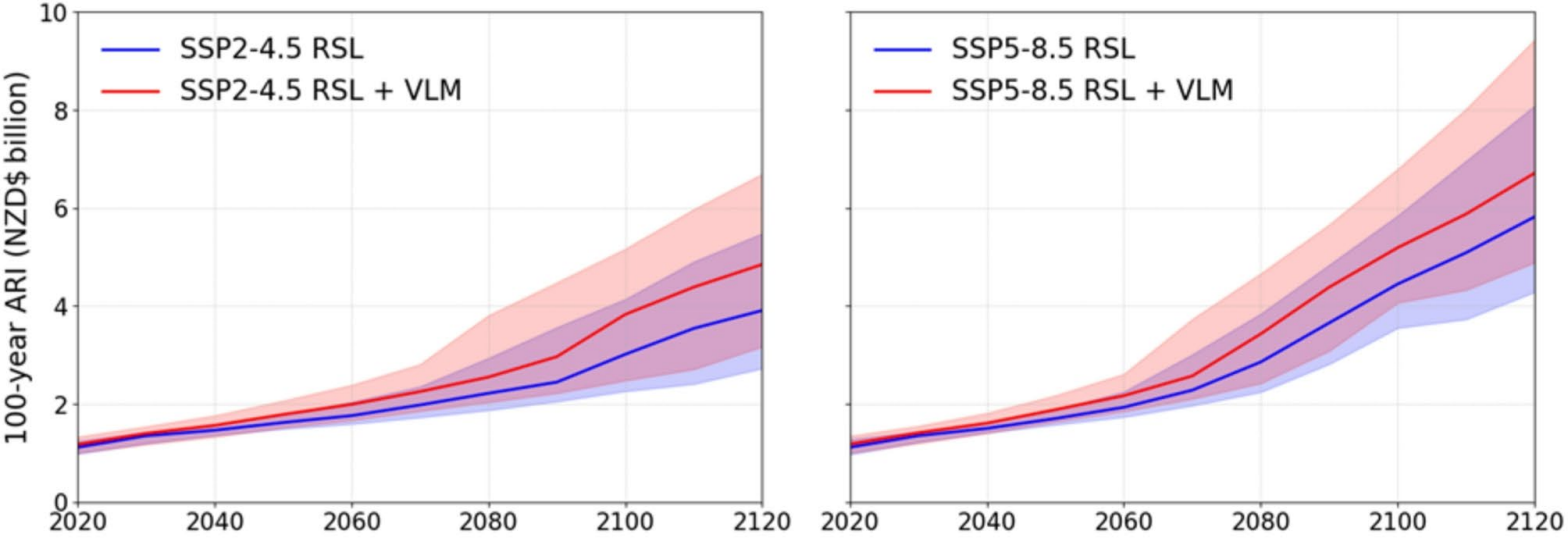
National building 100-year annual recurrence interval (ARI) loss estimated for episodic coastal flooding under RSL projections to 2120 for median and likely (17th-83rd percentile) GMSL range estimated for processes known with medium confidence for SSP2-4.5 and SSP5-8.5.

Financial losses in New Zealand from episodic coastal flooding show an acceleration later this century. Total potential losses to 100-year ARI flooding at present-day (i.e., year 2020) are estimated at NZD$1.3 billion (1.1–1.6). Under GMSLR alone losses could increase by NZD$1.6 billion (0.9–2.8) and NZD$3.3 billion (2.4–4.7) between 2050 and 2100 for SSP scenarios 2-4.5 and 5-8.5 respectively (Fig. 1). Local VLM may further increase losses by 15% and 10% respectively. This shortens the timing of losses expected from GMSLR alone by 10–12 years at 2100. After 2100, relative difference between losses under GMSLR and GMSLR with VLM reduces to 5%. This indicates accelerating GMSLR will exceed constant local VLM rates to become the more dominant driver of building losses in the next century.

**Fig. 2:**
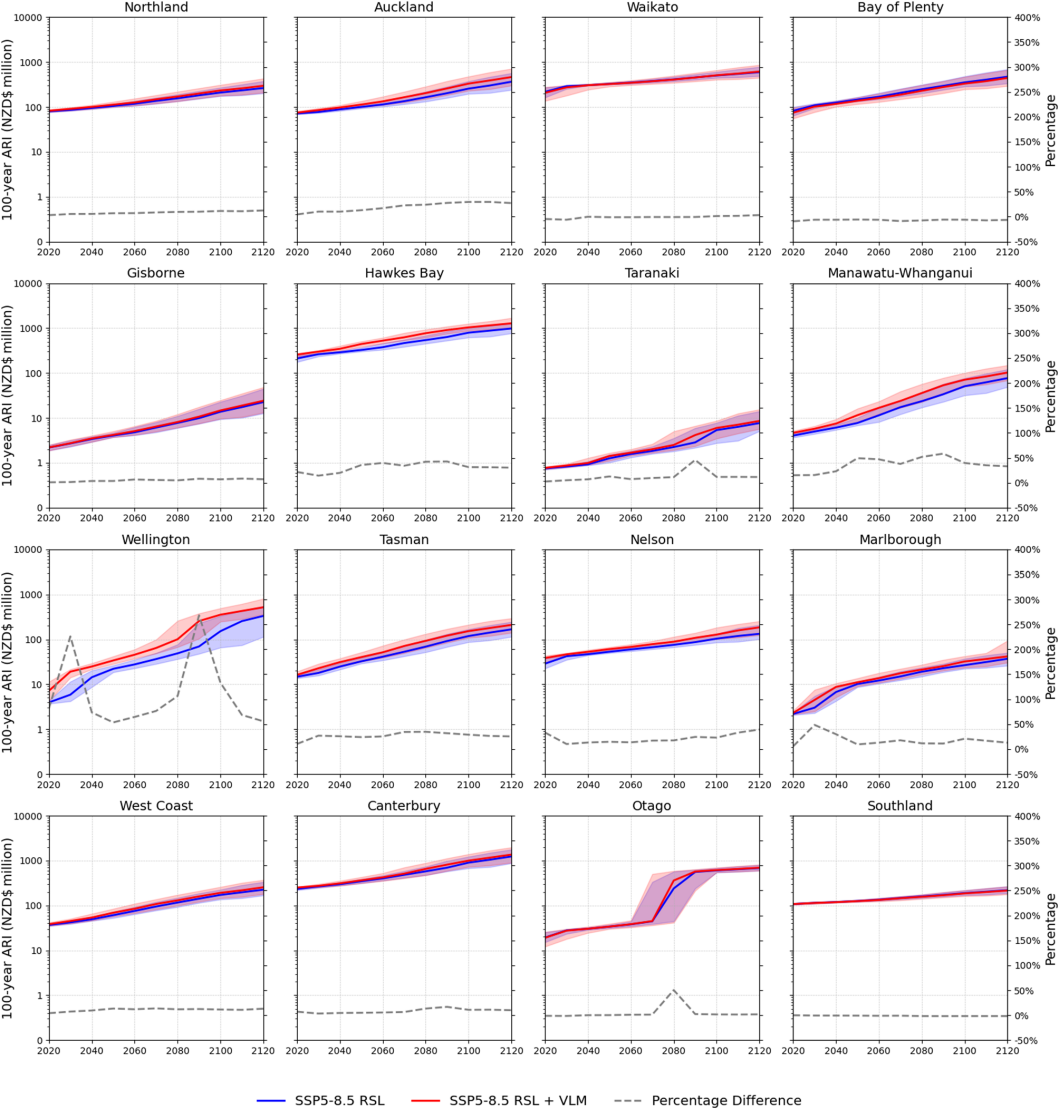
Regional building 100-year annual recurrence interval loss estimated for episodic coastal flooding under RSL projections to 2120 for median and likely (17th-83rd percentile) GMSL range estimated for processes known with medium confidence for SSP5-8.5.

Local VLM becomes a dominant factor in regional financial loss timing and magnitude for 100-year ARI flooding towards the end of this century. Several populous regions (i.e., Auckland, Hawkes Bay, Wellington and Canterbury) with downward land motion observed median losses at 2050 between 5% and 47% higher than GMSLR alone, increasing to 10% and 124% at 2100 under SSP5-8.5 scenarios (Fig. 2). These proportional differences at regional-level are highly variable under rising RSLs. In the Wellington region, subsidence rates exceeding 2 mm yr^−1^ VLM^21^ resulted in 235% (NZD$186 million (56–148)) higher losses in 2090. The VLM effect brings forward 100-year ARI flooding loss timing by up to 20 years between 2050 and 2100 compared to GMSLR alone. A similar timing shift occurs in the Hawkes Bay region. Main population centres in these regions are located on low-lying and subsiding land boarding estuarine environments^22^. This presents a significant concern for coastal resource managers whereby subsidence will accelerate decreasing design levels for structures (e.g., levees, seawalls, flood gates) affording flood protection after 2050. Under SSP5-8.5 scenarios, upward VLM exceeding > 2 mm yr^−1^ in the Bay of Plenty region reduced losses by 6% (2–14%) at 2100 compared to GMSLR alone. Upward VLM motion causes regional 100-year ARI flood losses at 2100 to occur up to 10 years later, than losses expected from GMSLR.

### Vertical land motion effects on average annualised loss

**Fig. 3:**
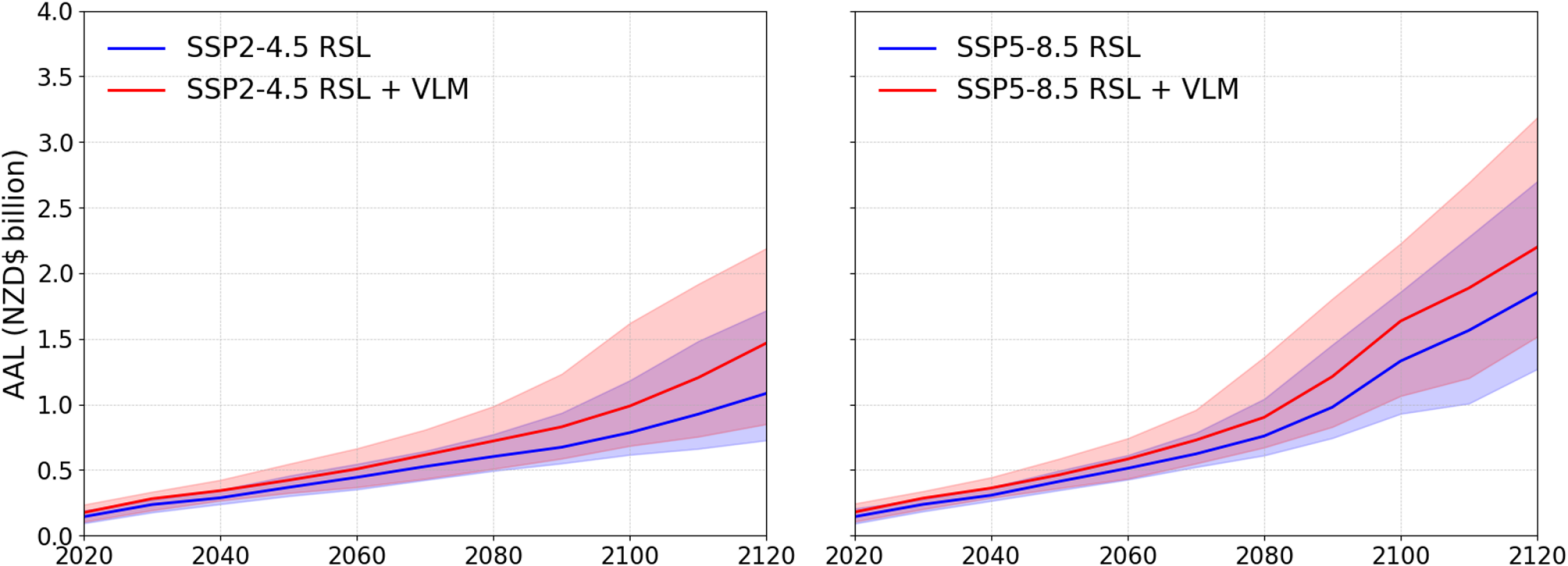
National building average annual loss estimated for episodic coastal flooding under RSL projections to 2120 for median and likely (17th-83rd percentile) GMSL range estimated for processes known with medium confidence for SSP2-4.5 and SSP5-8.5.

Episodic flooding by the end of this century could cause national annual losses between NZD$0.9 billion (0.7–1.4) and NZD$1.5 billion (1.1–2.2) under SSP2-4.5 and SSP5-8.5 scenarios respectively (Fig. 3). Downward land motion shortens the expected timing of AAL change over the next century. Compared to GMSLR alone, the projected AAL at 2100 is NZD$243.6 million (81.6-524.2) and NZD$365.3 million (162.8-442.2) higher respectively when VLM is also considered. This results from downward land motion increasing AAL between 10 and 22% from 2050 to 2100 compared to GMSLR alone. The downward motion effect would cause GMSLR projected AAL at 2100 to occur 15 years earlier for SSP2-4.5 and 11 years earlier for SSP5-8.5. This effect on expected AAL timing reduces after 2100 as GMSLR accelerates next century^20,23^.

**Fig. 4:**
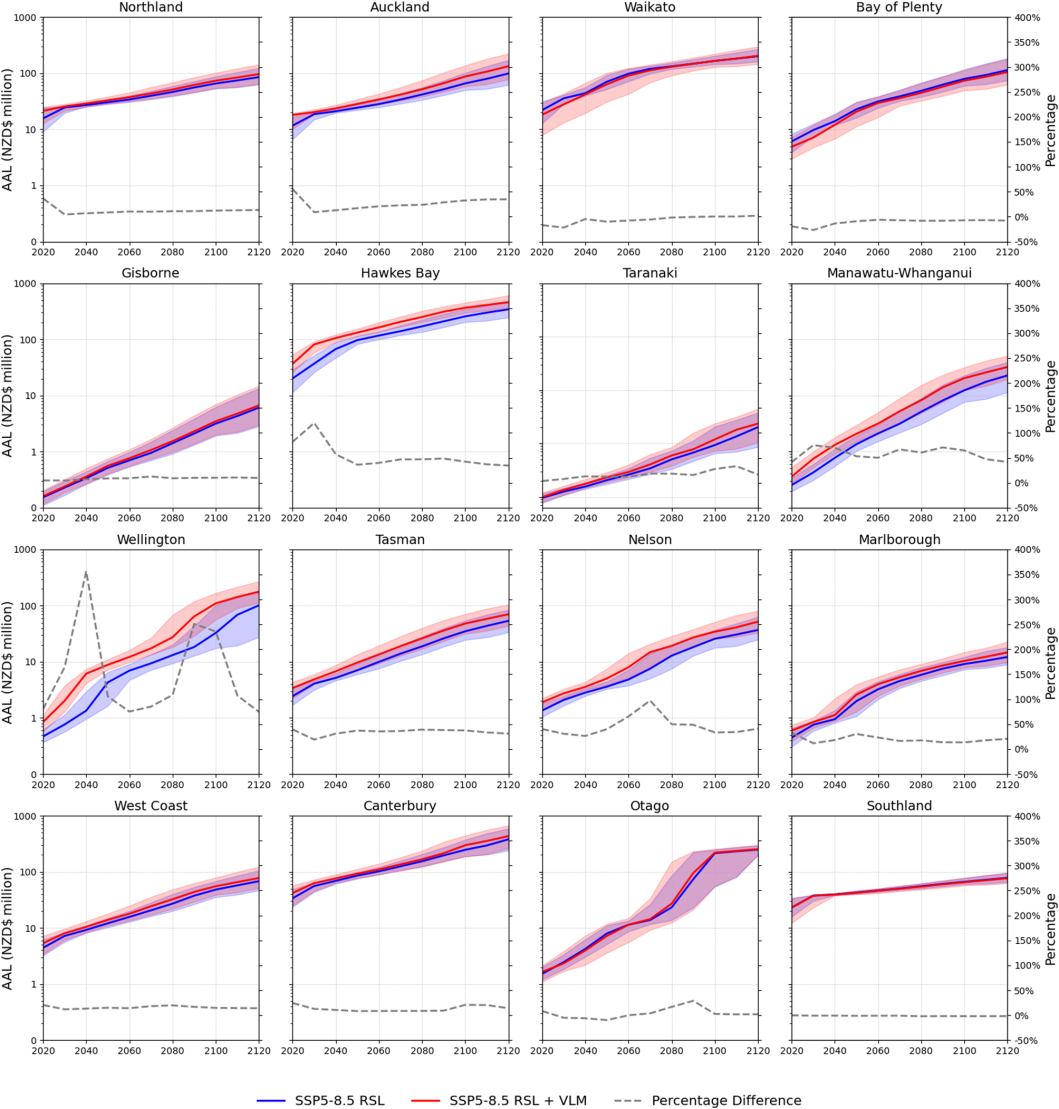
Regional building average annual loss estimated for episodic coastal flooding under RSL projections to 2120 for median and likely (17th-83rd percentile) GMSL range estimated for processes known with medium confidence for SSP5-8.5.

Local vertical land motion effect on RSL can shorten or lengthen regional AAL timing over the next century by 3 to 25 years. Regions with high > 2 mm yr^−1^ downward land motion could expect AALs projected under GMSLR alone to occur decades earlier after 2050 (Fig. 4). In Hawkes Bay region, downward VLM under SSP5.8-5 causes the expected AAL at 2050 to be NZD$35 million (31.6–39.3) higher than losses from GMSLR alone. In Wellington region downward VLM accelerates rapidly after 2080 with the expected GMSLR-driven AAL at 2100 occurring 18 years earlier. Regions with small AAL differences (< 20%) between these RSL scenarios showed AAL acceleration over this century is slowed by upward land motion. In Bay of Plenty and Waikato regions delays future AAL timing by 7–10 years in the decades between 2050 and 2100. In Waikato region the combined effects of downward land movement and GMSLR after 2100 accelerates AAL increase, whereas upward movement in Bay of Plenty region continues to limit AAL increase from GMSLR.

**Fig. 5:**
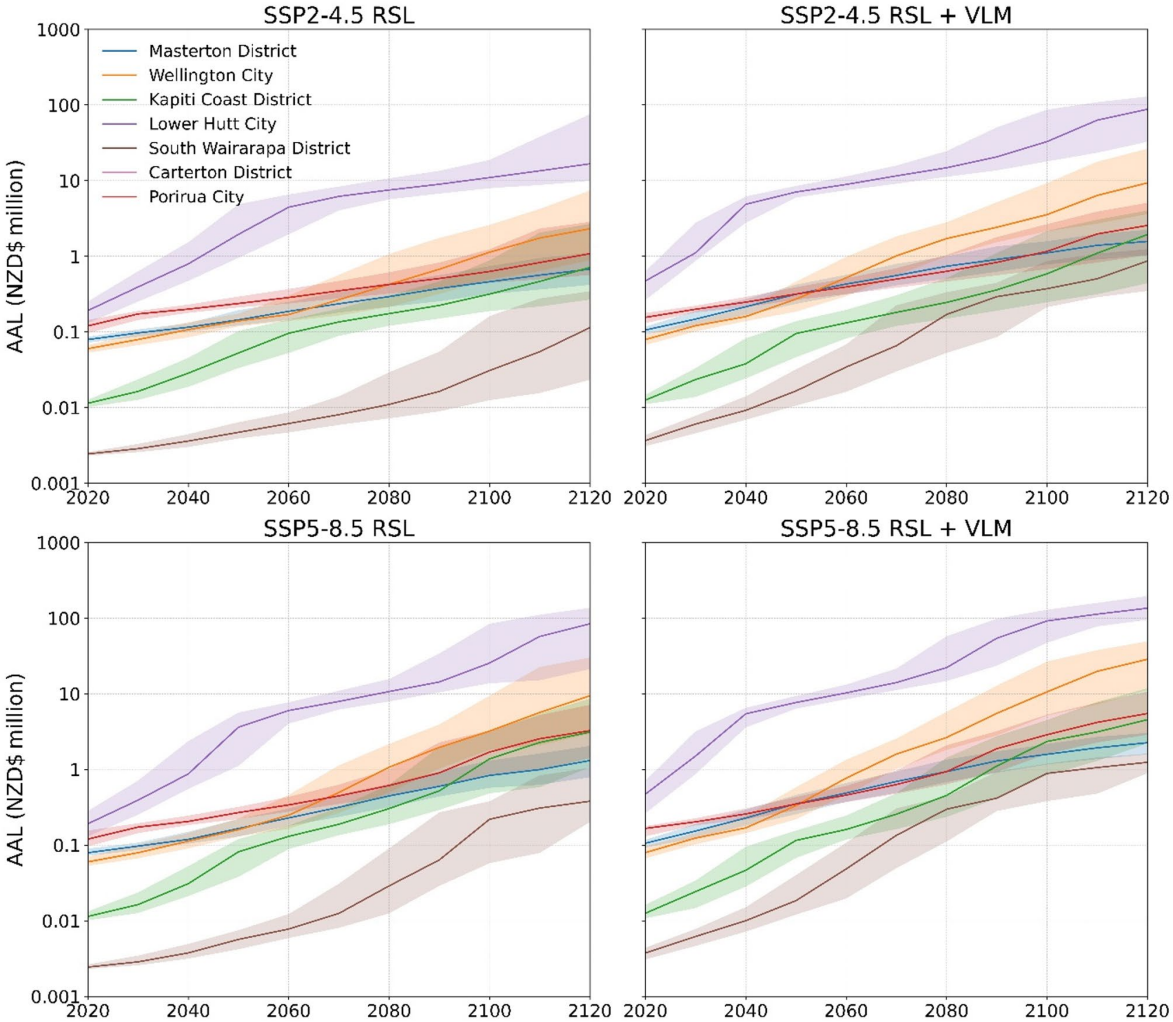
Wellington region territorial authority building average annual loss estimated for episodic coastal flooding under RSL projections to 2120 for median and likely (17th-83rd percentile) GMSL range estimated for processes known with medium confidence for SSP5-8.5.

**Fig. 6:**
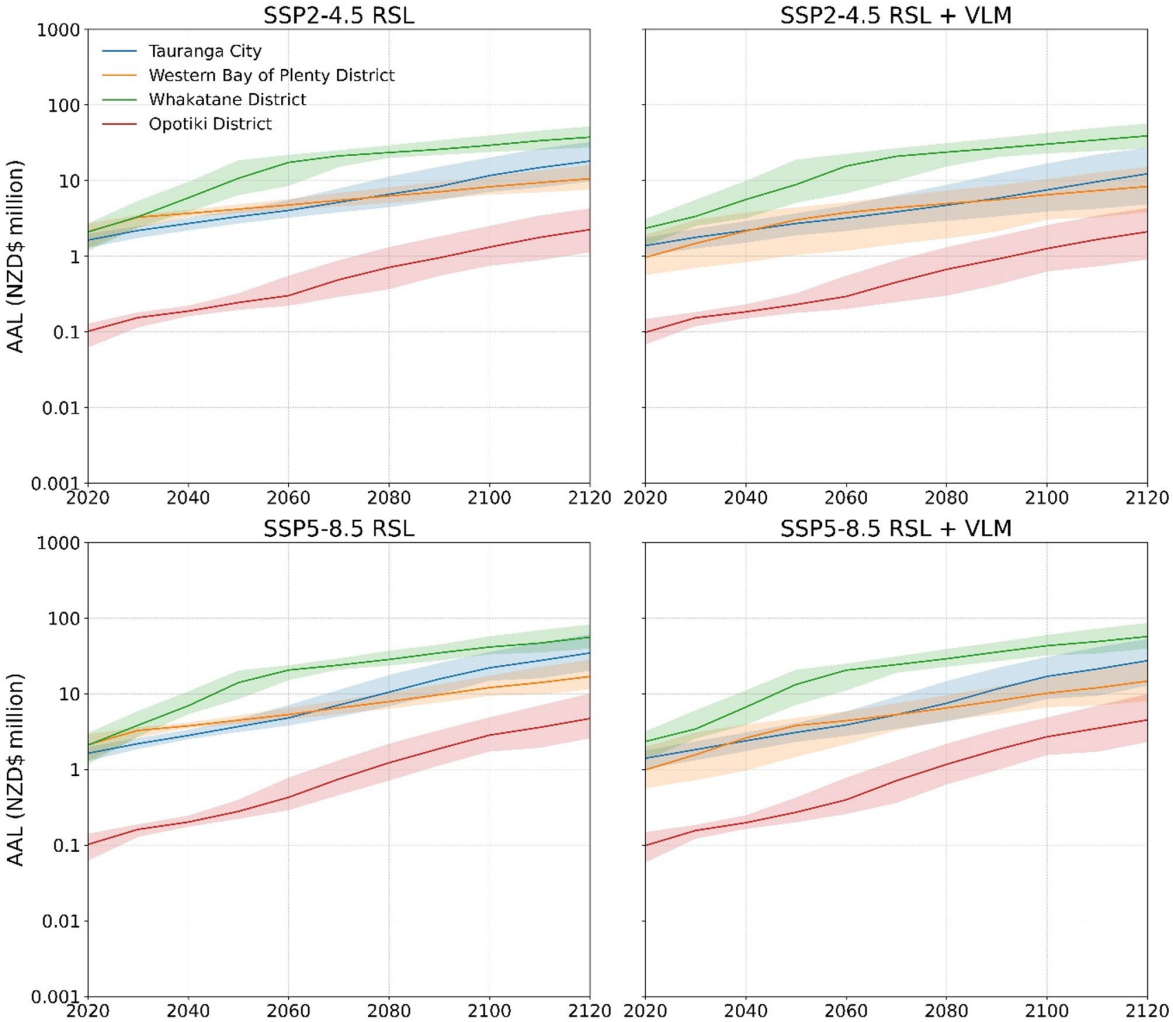
Bay of Plenty region territorial authority building average annual loss estimated for episodic coastal flooding under RSL projections to 2120 for median and likely (17th-83rd percentile) GMSL range estimated for processes known with medium confidence for SSP5-8.5.

Coastal flood risk models at ‘micro-levels’ (i.e., urban area, coastline segment) evaluate and can influence cost-efficient flood risk mitigation strategies and interventions^15^. In this study, building-level evaluation facilitated financial loss enumeration at different jurisdictional levels (i.e., national, regional and territory). Over large geographical areas, this approach avoided masking local VLM effects on AAL timing and magnitude over the next century. Here, AAL changes for SSP2-4.5 and 5-8.5 scenarios are presented for territories in Wellington (Fig. 5) and Bay of Plenty (Fig. 6) regions. Rapid downward or upward land motion > 2 mm yr^−1^ occurs in major urban areas (i.e., population > 100,000) for these regions^24^. In Lower Hutt City (Wellington region) AAL increases by 39% (SSP2-4.5) and 119% (SSP5-8.5) between 2080 and 2100 due to GMSLR. Local downward land movement over this period more than doubles AAL rates to 98% and 265%, shortening GMSLR estimated AAL at 2100 by ~ 25-years. Lower Hutt City will comprise over 80% of Wellington region’s coastal flood risk by 2100 in response to downward land movement. In the Bay of Plenty region, upward land motion in Tauranga City extends GMSLR estimated AAL at 2100 by 19-years 10-years under SSP2-4.5 and SSP5-8.5 respectively. Slower upward land motion (< 1 mm yr^−1^) limits this effect in Whakatane District with minimal difference in AAL timing for RSL scenarios over the next century. These coastal flood risk trends emphasise the importance for downscaling risk models to spatiotemporal resolutions compatible for evaluating financial losses in response to local RSL changes over the next century.

### Study limitations

This study has important limitations to be recognised. The results are difficult to compare with financial losses from historic coastal flood events or analytical studies in the local context. Major coastal flooding events during the 20th century^25^ have occurred in New Zealand during periods when official records on building-related financial losses are limited. Evaluating model uncertainties in future damaging flood events requires building-level investigation using dynamic models^26^ that simulate site-specific hazard conditions, coupled with observed building damage to quantify financial loss accuracy in this study. Present-day coastal flooding hazards and building exposure are represented in a future RSL change context. New Zealand has followed global trends over the past few decades with rapid urban development and population growth on low-lying coastal land^17,27^. Climate change and social factors will drive economic risks from episodic flooding over the next century^28^. Economic risk will increase in developed coastal areas due to population migration in response to socio-economic development opportunities^29,30^. Nationwide spatiotemporal risk profiling at different jurisdictional levels identifies priority areas for evaluating future growth scenarios and risk as coastal floodplains change under GMSLR and local VLM. The present study is limited to direct building financial losses therefore, future methodology iterations should include a broader built-asset (e.g., infrastructure network components, agriculture) range for economic risk evaluation. This advancement facilitates a more comprehensive risk and cost-benefit analysis of optimal flood mitigation interventions for coastal communities.

## Conclusion

This nationwide study estimated financial losses for extreme sea level (ESL) driven flooding and relative sea level (RSL) change over a future 100-year period. Financial losses were evaluated at the building asset-level using a coastal flood risk model framework in RiskScape. The model calculated time-independent financial losses from nine annual recurrence interval (ARI) ESLs and twenty-one MSL scenarios. A national RSL projections database was used to evaluate loss changes over the next 100-years in response to global mean sea level rise (GMSLR) and local vertical land motion (VLM).

Financial loss timing and magnitude for 100-year ARI and AAL was reported at national and sub-national levels, focusing on Shared Socio-economic Pathways (SSP) 2-4.5 and 5-8.5 medium confidence scenarios. Local VLM may increase national 100-year ARI losses by 15% and 10% at 2100 compared to GMSLR alone, shortening the timing of potential losses by 10–12 years. Downward or upward land motion shortens or lengthens regional GMSLR-driven AAL at 2100 by 3 to 25 years. Larger projected AAL timing differences occurred in regions with > 2 mm yr^−1^ VLM. Our findings in Wellington and Bay of Plenty regions demonstrated that high VLM rates within populous urban areas shift financial loss timing and magnitude by decades over the next century. Such local effects were masked in projected national AAL estimates over the next century. Our study has demonstrated local VLM influence on financial loss timing and magnitude for episodic coastal flooding over the next century and requires consideration when evaluating coastal flood mitigation interventions under changing RSLs.

## Methods

### Risk model

The model workflow used in this study was centred on a conceptual framework for risk quantification:

$$\:R={f}_{C}({H}_{h},\:E,{V}_{h})$$

where risk (*R*) is a function ($\:{f}_{C}$) of the consequences from an ESL event (*H*) impacting an exposed (*E*) building. Consequences are determined from building vulnerability (*V*) to a damage type (e.g., financial loss) and magnitude in response to ESL event (*h*). We implement the coastal flood risk model workflow in RiskScape^29,30^a general-purpose multi-hazard risk modelling software. RiskScape’s modular framework was well suited to evaluate financial losses from coastal flooding under present and future climates. Model input parameters are represented by hazard, exposure and vulnerability data derived from external models, and processed in the risk model workflow using geospatial or statistical sampling functions. Building structure damage financial losses are estimated for ESL coastal flooding scenarios and enumerated for national, regional and territory levels. Details on the analysis components are provide below.

### Extreme sea level model

Nationwide episodic flooding from extreme sea-levels (ESLs) were available in this study for present-day and future higher sea levels^31^. ESL flooding maps represent 2, 5, 10, 20, 50, 100, 200, 500 and 1,000 annual recurrence intervals (ARI). ESL was calculated linearly from several coastal processes:

$$ESL=MSL+ST+WS+RSL.$$

where MSL is mean sea level relative to local vertical datum calculated from sea-level gauge records over (approximately) a recent decade, ST is the storm-tide combination of high tide, meteorological effects (storm-surge) and monthly sea-level anomaly, affected by both seasonal heating and cooling and interannual and inter-decadal climate variability such as the El Niño Southern Oscillation (ENSO), WS is the additional wave setup (over and above ST) at the shoreline where breaking waves are present, and RSL is relative sea level change.

ESL flooding maps were created using a static inundation approach^32^ to identify topographical elevations below ESL elevations. Inundation was mapped on a composite digital elevation model (DEM) for coastal land up to 20 m above present-day mean sea levels^31^. The national DEM was created from local LIDAR DEMs resampled to 10 m resolution using a nearest-neighbour interpolation, combined with the 1 arc-second (30 m) Shuttle Radar Topography Mission (SRTM)^33^ DEM. SRTM represented coastal land without a LIDAR DEM. The vertical bias created by STRM was improved using a fully convolutional neural network (FCN) model^34^. Water depth above ground was computed for DEM grid cells from the difference between ESL water surface height and land elevation. Grid cells without a hydrologic connection to coastlines were removed to limit inundation extent overestimation. Topographic protection structures (i.e., levees) were obtained from a national database^35^or where missing were digitised as polylines where identifiable from aerial imagery. Structure design-level absence required a uniform land protection level up to 100-year ESLs at present-day MSL (i.e., ESL + MSL = 0 m). ESL ARI flooding maps were produced for 0.1 m MSL increments up to 2 m (i.e., ESL + MSL = 0.1–2 m), independent of future RSL projections.

Global sea level change rates and timing is uncertain, especially from 2050 onwards^23^. Vertical land motion (VLM) at local scales can further exacerbate or reduce RSL changes^35^. Here, future RSL rates and timing around New Zealand’s coastline for various Shared Socioeconomic Pathways (SSPs) are based on probabilistic RSL projections^19,20^ developed under the Framework for Assessing Changes to Sea-level (FACTS) from IPCC Assessment Report 6^36^. RSL projections include VLM effects, derived from interferometric synthetic aperture radar (InSAR) data, calibrated with campaign and continuous Global Navigation Satellite System (GNSS)^24^. Projections over a 100-year period from 2020 to 2120 were downloaded from https://searise.takiwa.co/ for 7435 coastal sites. Probabilistic RSL curves (17th, 50th and 83rd percentiles) representing *medium confidence* climatic processes for each SSP scenario were spatially to joined to individual buildings to evaluate the future timing and uncertainty of future financial losses.

### Building exposure model

The exposure model estimated the replacement value of individual buildings exposed to ESL inundation. Building replacement value ($\:{B}_{R}$) was calculated using the general formula:

1 $$\:{B}_{R}=\sum\:_{i=1}^{n}\sum\:_{j=1}^{{m}_{i}}{C}_{Rij}\bullet\:\:{B}_{A}$$

where for building $\:i$, $\:{B}_{A}$ is the area (m^2^, $\:{C}_{R}$ is the replacement value for structure, external finishes, internal finishes, and service components ($\:{m}_{i}$) and *n* equals the number of components used to calculate the replacement value. B_R_ was computed as the sum of *i* = 1…*n*, enumerated from corresponding *j* = 1…*m* sub-component unit cost rates (NZD) determined from 2023 first quarter national building construction valuation guidelines^37^. Building sub-component unit costs were estimated based on each building’s geographical region, area (m^2^, primary use, number of storeys, structural frame and wall cladding (Table 1). Unit cost rates represented a median $\:{C}_{R}$ for *B*_*R*_ enumeration.

A national dataset representing 3,288,414 buildings^38^ with roof outline (i.e., building footprint) areas ≥ 10 m^2^ was used for this study. Several geometric and non-geometric attributes were considered in this study for building replacement valuation or damage model applications, including area (m^2^, primary use, number of levels (i.e., storeys), structural frame and wall cladding (Table 1). These attributes and floor heights were extracted from nationwide datasets^17,22,39^ representing coastal and fluvial floodplain buildings. Building attributes were spatially joined to buildings exposed within the maximum modelled ESL inundation area (i.e., 1000-year ARI + 2 m RSL).

**Table 1:** Building attributes considered for this study.

Attributes		Attribute classes or description	Data Type*
Location	Region	Northland; Auckland; Waikato; Bay of Plenty; Gisborne; Taranaki; Manawatu-Whanganui; Hawkes Bay, Wellington; Tasman; Nelson; Marlborough; Canterbury; West Coast; Otago; Southland	Text _(N)_
Geometric	Area	Building roof outline area (m^2^)	Integer _(C)_
	Floor Height	First finished floor height above ground level (m)	Decimal _(C)_
	Storeys	Number of complete building floor levels	Integer _(C)_
Non-geometric	Construction Period	< 1900; 1900–1920; 1920–1940;1940–1960; 1960–1980; 1980–2000; >2000	Text _(O)_
	Primary Use	Agricultural; Appurtenant; Commercial; Community; Cultural; Education; Emergency Service; Government; Industrial; Lifeline Utility; Residential	Text _(N)_
	Replacement Value	Building replacement value estimated in 2023 New Zealand dollars (NZD)	Integer _(C)_
	Structural Frame	Advanced Design; Brick Masonry; Concrete Masonry; Light Industrial; Light Timber; Reinforced Concrete Shear Wall; Reinforced Concrete Moment Resisting Frame; Steel Braced Frame; Steel Moment Resisting Frame; Tilt-Up Panel	Text _(N)_
	Wall Cladding	Brick masonry; Concrete masonry; Fibre-cement; Fibrolite; Mixed material; Roughcast; Sheet metal; Weatherboard;	Text _(N)_

*C Continuous, *N* Nominal, *O* Ordinal.

### Damage and loss model

Financial losses from building structure damage were estimated using the unit loss method. Here, financial loss (*L*) is calculated using the formula:

2 $$\:L=\:{B}_{Ri}\:{f}_{vi}\left({D}_{ij}\right)$$

where damage for building *i* is enumerated from water depths (*D*) at grid locations *j*, determining the damage ratio (i.e., ‘cost-to-repair’/’cost-to-replace’) from a corresponding building category depth-damage function ($\:{f}_{v}$(*D*)) to multiply by $\:{B}_{R}\:$to calculate *L*. Building structure damage analysis implemented a function suite^40^ representing flood damage responses for common New Zealand building classes based on their construction year, floor height, primary use, storeys and structural frame attributes (Table 1). The RiskScape engine executed depth-damage functions using conditional statements to apply class-specific functions based on building *i* attributes.

Spatiotemporal changes to financial loss occurrence were calculated as average annualised loss (AAL), also known as expected annual damage (EAD). The AAL is commonly used in the insurance sector to represent financial loss expected per-year from hazard occurrences^15^. Financial loss was calculated in this study for each ESL event to estimate the exceedance probability loss (EPL):

3 $$\:EPL={\sum\:}_{i=1}^{N}L\left(P\right)$$

where $\:L\left(P\right)$ is the financial loss annual probability of occurrence estimated for flood event *h* annual recurrence interval and *N* represents the total buildings affected by event *h*. A hypothetical loss curve is formed between $\:P$ and $\:\text{E}\text{P}\text{L}$ with a positive monotonic trend in response to decreasing $\:P$. The expected AAL is then estimated using trapezoidal integration to compute the area under the curve:

4 $$\:\text{A}\text{A}\text{L}={\int\:}_{{P}_{\text{m}\text{i}\text{n}}}^{{P}_{\text{max}}}EPL\left(P\right)$$

where $\:{P}_{\text{m}\text{a}\text{x}}$ represents the highest hazard event occurrence probability and $\:{P}_{\text{m}\text{i}\text{n}}$ represents the lowest event occurrence probability. $\:EPL\left(P\right)$ is the enumerated financial loss for an ESL event with the probability of occurrence $\:P.$ AAL was then estimated by solving the integral in Eq. 4 using the trapezoidal Riemann sum approach^40^.

### Methodological limitations

Several methodological limitations in this study must be acknowledged when interpreting results. The static inundation model approach, while computationally efficient at national scales, assumes instantaneous water level rise and inundation while neglecting dynamic coastal flood processes such as wave overtopping and run-up, tidal propagation, flow velocity and drainage^41,42^. As a result, under- or overestimation of local inundation extent and depths could over or under-state the regional and territorial building financial loss magnitude and timing. Further inundation extent and depth validation using calibrated dynamic models for is required to develop a more complete understanding modelled loss uncertainties across different coastal environments. Another limitation stems from constant VLM rates over the 100-year study period. While recent VLM rates derived from InSAR and GNSS data are valuable, they may change over time due to tectonic, anthropogenic, or isostatic processes. Assuming a linear trend may misrepresent future RSL change in areas of subsidence or uplift, leading to spatial biases in the projected financial loss timing and magnitude. Finally, ‘depth-damage’ functions, although widely adopted in large-scale flood risk assessments^3^simply building damage processes in coastal environments^43^. These functions predict physical damage from a constant water depth rise, without accounting for damage variability due to other flood hazard intensities (e.g., flow velocity, duration) physical and non-physical building characteristics and other phenomena (e.g., emergency preparedness). Future enhancements to damage estimation can be made by incorporating empirically derived multivariable models that facilitate a probabilistic representation of damage and loss uncertainty^44^.

## Data Availability

The datasets generated during and/or analysed during the current study are available from the corresponding author upon reasonable request. GIS data for New Zealand buildings is available at https://data.linz.govt.nz/layer/101290-nz-building-outlines/. New Zealand relative sea level rise projections for Shared Socioeconomic Pathways (SSPs) are available at https://searise.takiwa.co/. 100-year extreme sea level flooding maps for a 100-year annual recurrence interval event and relative sea level rise are available at https://niwa.co.nz/hazards/coastal-hazards/extreme-coastal-flood-maps-aotearoa-new-zealand.
